# Load Regulates Bone Formation and Sclerostin Expression through a TGFβ-Dependent Mechanism

**DOI:** 10.1371/journal.pone.0053813

**Published:** 2013-01-07

**Authors:** Jacqueline Nguyen, Simon Y. Tang, Daniel Nguyen, Tamara Alliston

**Affiliations:** 1 Graduate Program in Oral and Craniofacial Sciences, University of California San Francisco, San Francisco, California, United States of America; 2 Department of Orthopaedic Surgery, University of California San Francisco, San Francisco, California, United States of America; 3 Department of Bioengineering and Therapeutic Sciences, University of California San Francisco, San Francisco, California, United States of America; 4 Department of Otolaryngology Head and Neck Surgery, University of California San Francisco, San Francisco, California, United States of America; 5 Eli and Edythe Broad Center of Regeneration Medicine and Stem Cell Research, University of California San Francisco, San Francisco, California, United States of America; Medical College of Georgia, United States of America

## Abstract

Bone continually adapts to meet changing physical and biological demands. Osteoblasts, osteoclasts, and osteocytes cooperate to integrate these physical and biochemical cues to maintain bone homeostasis. Although TGFβ acts on all three of these cell types to maintain bone homeostasis, the extent to which it participates in the adaptation of bone to mechanical load is unknown. Here, we investigated the role of the TGFβ pathway in load-induced bone formation and the regulation of Sclerostin, a mechanosensitive antagonist of bone anabolism. We found that mechanical load rapidly represses the net activity of the TGFβ pathway in osteocytes, resulting in reduced phosphorylation and activity of key downstream effectors, Smad2 and Smad3. Loss of TGFβ sensitivity compromises the anabolic response of bone to mechanical load, demonstrating that the mechanosensitive regulation of TGFβ signaling is essential for load-induced bone formation. Furthermore, sensitivity to TGFβ is required for the mechanosensitive regulation of Sclerostin, which is induced by TGFβ in a Smad3-dependent manner. Together, our results show that physical cues maintain bone homeostasis through the TGFβ pathway to regulate Sclerostin expression and the deposition of new bone.

## Introduction

Osteocytes coordinate the adaptation of bone to changing physical demands on the skeleton [1]. Upon sensing mechanical load through their canalicular processes, osteocytes initiate a series of biochemical signaling events that coordinate the activity of osteoblasts and osteoclasts to increase bone mass [2]. In this way, physical stimuli employ established biochemical pathways long known to participate in the maintenance of bone homeostasis, including parathyroid hormone (PTH) [3], insulin-like growth factor-I (IGF-1) [4], and prostaglandin signaling (PGE_2_) [5]. Despite recent progress in deciphering the molecular mechanisms by which physical signals regulate bone homeostasis, many questions remain.

Sclerostin, a secreted protein expressed by osteocytes, responds to mechanical load and antagonizes bone formation [6]. Loss of function mutations in either the sclerostin-encoding gene, *SOST*, or in its regulatory sequence cause the human syndromes known as sclerosteosis and van Buchem disease, both of which are characterized by high bone mass [7]. Sclerostin acts by binding to the Wnt co-receptor Lrp5/6 on osteoblasts to inhibit Wnt-inducible osteogenesis [8]. Sclerostin plays a central role in the anabolic response of bone to mechanical loading. Applied mechanical loads repress Sclerostin mRNA and protein expression [6], thereby releasing the brakes on new bone synthesis. Conversely, Sclerostin-antagonizing antibodies prevent bone loss due to unloading of the bone [9]. Several pathways that control bone development and metabolism also regulate *SOST* expression. The BMP pathway induces *SOST* expression during bone development [10]. PTH, an essential regulator of mineral homeostasis, represses *SOST* expression [11]. This PTH-mediated repression of *SOST* requires MEF2 recruitment to a highly conserved regulatory region 35-kb downstream from the *SOST* gene [12]. The rapid increase in PGE_2_ following mechanical load also contributes to the mechanosensitive repression of *SOST*, though the mechanism remains to be identified [13]. The master osteoblast transcription factor Runx2 binds and induces transcription from a more proximal element of the *SOST* promoter [14]. Although many pathways modulate *SOST* expression, these pathways do not fully explain the complexity of mechanotransduction in bone and the regulation of *SOST* expression.

TGFβ is a critical regulator of bone homeostasis. Through its effects on osteoblast and osteoclast migration, proliferation, differentiation and viability, TGFβ couples bone formation with bone resorption [15], [16]. In this way, TGFβ maintains both bone mass and bone quality [17]–[18]. Activated TGFβ binds to its receptors, TβRI and TβRII, causing their heterotetramerization and transphosphorylation. Many intracellular proteins, including Smad2, Smad3 and other non-canonical effectors, are phosphorylated upon recruitment to the activated TβRI/TβRII complex [19]. Phosphorylated Smad3 translocates to the nucleus where it can activate or repress the activity of sequence-specific transcription factors, such as Runx2 [20]. Through crosstalk at each the ligand, receptor and effector levels, the TGFβ pathway integrates signals from multiple stimuli. For example, fluid flow induces TGFβ1 mRNA expression in SAOS-2 cells [21]. Cell-generated tension by myofibroblasts converts the TGFβ ligand from the latent to active form [22]. PTH receptors drive clustering and internalization of TβRI and TβRII, leading to desensitization of the pathway [23]. The nuclear localization of Smads is sensitive to other biochemical and physical cues, including Wnt signaling [24], cytoskeletal tension [25], and extracellular matrix stiffness [26]. However, the net effect of mechanical load on the overall activity of the TGFβ pathway remains unknown. Therefore, we hypothesize that the TGFβ pathway integrates signals provided by mechanical load to maintain bone homeostasis, in part by regulating the expression of Sclerostin.

Using a combination of genetically modified mouse models and *in vitro* approaches, we investigated the effect of mechanical load on TGFβ activity, the role of TGFβ in load-induced bone formation, and the regulatory relationship between TGFβ and Sclerostin. Taken together, our results suggest that TGFβ plays a critical role in the mechanosensitive regulation of Sclerostin and is required for the anabolic response of bone to mechanical load.

## Materials and Methods

### Mouse models

Treatments and protocols used for the animal studies were approved by the University of California, San Francisco Institution Animal Care and Use Committee (Protocol # AN082159-03A) and were designed to minimize discomfort to the animals. This study used 8–9 week-old male SBE-luciferase mice [27] or DNTβRII mice, which express a dominant negative version of TβRII under control of 1.8 kb of the osteocalcin promoter [28]. Wild type littermates were used as comparative controls.

### *In vivo* mechanical loading of the tibiae

Axial compressive loads equivalent to 10 times the mouse's body weight were delivered by a Bose Electroforce ELF3200 desktop load frame (Bose, MN, USA) fitted with two custom-made hemi-spherical fixtures that gripped the mouse knee and ankle. Similar methods of *in vivo* loading have been shown to upregulate bone anabolism [29]. In our preliminary studies of *ex vivo* limb loading using in situ strain rosettes, these loading parameters produce maximum principal strains in the range of 1500–2500 με on the mid-diaphyseal surface of the tibiae. For each mouse, only the right hind limb was loaded, while the left hind limb was not loaded to serve as the contralateral control (nonloaded). Each round of loading consisted of 600 cycles of axial compression at 1 Hz administered under general injectable anesthesia. Mice were subjected to this loading regimen once daily for either 1 day (short-term) or once a day for 5 days (prolonged).

### *In vivo* luciferase imaging

Five hours after a single bout of loading, the SBE-luc mice (n = 6) were anesthetized using isoflurane, injected with 150 mg D-luciferin (Xenogen) per kg body weight, and imaged using the IVIS-200 Bioluminescence system 10 minutes after injection (Caliper Bioscience, MA, USA) [22], [30]. Photons emitted from living mice were acquired as photons per second per cm^2^ per steradian (sr) by using LIVINGIMAGE software (Xenogen) and integrated over 20 min. For photon quantification, a region of interest was manually selected and kept constant within all experiments; the signal intensity was converted into photons/s/cm^2^/sr. The resulting quantitative measures were segmented and contoured at the tibiae to determine the relative levels of Smad2/3 activity between the loaded and non-loaded limb.

### Immunohistochemistry

The loaded and nonloaded tibiae from SBE-luciferase (n = 5), DNTβRII (n = 5), or WT mice (n = 5) were dissected and fixed in 2% paraformaldehyde for 24 h at 4°C. The bones were decalcified in 19% EDTA solution for 7–10 days and decalcification was confirmed with x-ray imaging. Bones were then infiltrated with a series of 10%, 20%, and 30% sucrose solutions [31]. After sucrose infiltration, each tibia was cut into three fragments, with the distal fragment being 8 mm long and the middle fragment being 6 mm long, to ensure that comparable sections of each region were analyzed for each bone. Each fragment was embedded in OCT for frozen sectioning (10 μm sections). The tissue sections were permeabilized with 0.3% Triton X-100, processed for antigen retrieval with Ficin (Invitrogen 00–3007), and blocked for intrinsic peroxidase activity with 3% hydrogen peroxide. For detection of luciferase, the sections were blocked with 1.5% normal goat serum (Vectastain) and incubated with anti-Luciferase primary antibodies (Abcam ab21176) at a dilution of 1∶100. For detection of Sclerostin, the sections were blocked in 1.5% normal rabbit serum (Vectastain) and incubated with anti-Sclerostin primary antibodies (R&D Systems AF1589) at a dilution of 1:13 with 0.05% Tween-20. Normal rabbit IgG (Caltag Lab 10500) and normal goat IgG (Santa Cruz sc-2028) were used at the same concentrations as primary luciferase and Sclerostin antibodies, respectively, to control for the specificity of immunostaining. The binding of peroxidase-conjugated secondary antibodies was detected with a DAB kit (Vector Lab).

Three to five immunostained sections for each bone were analyzed quantitatively. A composite of 20X images was generated for each immunostained section, from which all DAB-stained (brown) and unstained lacunae were counted and recorded using ImageJ. The percent of osteocytes expressing luciferase or Sclerostin was determined by dividing the number of DAB-positive lacunae by the total number of lacunae for each section. The percent change in luciferase or Sclerostin expression due to load was determined by subtracting the averaged percentage of luciferase or Sclerostin expression in the nonloaded tibia from the loaded tibia as describe [6]. Since quantitative analyses consider the effect of loading only on the number of stained and unstained lacunae, not on the staining intensity, they likely underrepresent the effects of mechanical loading on Sclerostin and luciferase expression.

### Micro-computed tomography

Five hours after the last session of *in vivo* loading of the DNTβRII mice and their WT littermates (n = 6), tibiae were dissected from euthanized mice, fixed in 4% paraformaldehyde in PBS, and serially dehydrated in graded ethanol. The bones were then scanned using micro-CT to determine the relative changes in bone geometry (VivaCT40, Scanco Medical AG). The micro-CT scanner was operated at the peak energy of 70 kVp, current of 114 μA, integration time of 381 ms, and a 10 μm voxel resolution. The scans were segmented using an attenuation constant of 200, and then the structural parameters of bone were evaluated. The proximal tibial trabecular bone was evaluated for changes in trabecular connectivity, trabecular thickness, and volume fraction. The cortical bone changes were evaluated at the tibio-fibular junction for cortical thickness, cross-sectional area, and moment of inertia.

### Dynamic histomorphometry

Two intraperitoneal injections of calcein were administered at 0.02 mg/g body weight to DNTβRII mice and their WT littermates (n = 3) that underwent a prolonged 5-day loading regimen. The first injection was administered on the same day as the first loading bout, and the second injection was administered on the same day as the fifth and final loading bout (4 days apart). The mice were euthanized two days after the final loading bout and then the tibiae were collected and fixed in 4% paraformaldehyde in PBS, serially dehydrated in graded ethanol, and embedded in a plastic resin (TechnoVit 5, EMS, Pennsylvania). The embedded blocks were sectioned using a tungsten carbide blade and mounted on glass slides. The mineral apposition rate (MAR, mean inter-label thickness divided by the time between the two labeling periods) was computed at both the periosteal and the endosteal surfaces using ImageJ by evaluating fluorescent micrographs taken with a 20X optical objective. The Mineral Apposition Rate (MAR) was calculated in accordance with the ASBMR nomenclature [32].

### Cell culture

UMR-106 cells (ATCC), a rat osteosarcoma cell line, and SAOS-2 cells (ATCC), a human osteosarcoma cell line, were used for the *in vitro* studies because they both express Sclerostin [12] [14]. UMR-106 cells were grown in DMEM 50% high glucose/50% F-12 media with 10% fetal bovine serum supplemented with 1% nonessential amino acids. SAOS-2 cells were grown in McCoy's 5A media with 15% fetal bovine serum. At 80% confluence, UMR-106 or SAOS-2 cells were treated with 5 ng/ml TGFβ1 (Peprotech, 100–36E) or 10 μM SB431542 (Sigma, S4317) for the indicated times. Cycloheximide (10 μg/ml, Sigma) or actinomycin D (1 μg/ml, Sigma) were added to cells 30 min prior to TGFβ treatment. For *Runx2* knockdown in SAOS-2 cells, cells were transiently transfected with 50 nM of human *Runx2* siRNA r(GGUUC AACGAUCUGAGAUU)d(TT) or AllStars negative control using Qiagen Hi-Perfect. For *Runx2* knockdown in UMR-106 cells, cells were transfected with 60 nM of rat *Runx2* siRNA (GACUCUAAACCUAGUUUGU[dt][dt]) and *Runx2* siRNA AS(ACAAACUAGGUUUAGAGUC[dt][dt]) (Sigma) or Sigma siRNA negative control (Sigma sic-001) using Qiagen Hi-Perfect. SAOS-2 cells were treated with TGFβ1 (5 ng/ml) for 24 h and cells were harvested 48h after transfection. UMR-106 cells were transfected for 72 h with repeated transfection every 24 h. At the last transfection, UMR-106 cells were treated with 5 ng/ml TGFβ1 and harvested 24 h later. All results shown are from UMR-106 cells, but they were repeated in SAOS-2 cells with similar but not identical conclusions.

### RNA extraction and quantitative PCR

RNA was extracted from cells using the Qiagen RNeasy Mini Kit. cDNA was generated from up to 1 μg of RNA with the iScript cDNA Synthesis kit (Bio-Rad). Quantitative PCR was performed using iQ SYBR Green Supermix (Bio-Rad) with the following primers: rat *L19*-F (GCATATGGGCATAGGGAAGA); rat *L19*-R (CCATGAGAATCCGCTTGTTT); rat *SOST*-F (GCACCATGCAGCTCTCATTA); rat *SOST*-R (CATTCTTGAAGGCTTGCCAC); rat Runx2-F (ACCCAGGCGTATTTCAGATG); rat Runx2-R (AGTGAGGGATGAAATGCCTG); human *L19*-F (GGGATTTGCATTCAGAGATCAG); human *L19*-R (GGAAGGGCATCTCGTAAG); human *SOST*-F (GGACTCCAGTGCCTTTTGAA); human *SOST*-R (CTGAATTCTGGAAGTGACCTTG); human *Runx2*-F (CCCCACGACAACCGCACCAT); human *Runx2*-R (CACTCCGGCCCACAAATC).

### Western Analysis

For Western analysis of cortical bone protein, both tibiae were collected 3–5 h after one application of unilateral load (n = 4). Bones were trimmed to remove the distal and proximal epiphyses and flushed with PBS to remove bone marrow. The remaining cortical bone was homogenized with a rotor-stator homogenizer (Omni) in radioimmunoprecipitation assay (RIPA) buffer (10 mM Tris-HCl pH 8, 140 mM NaCl, 1 mM EDTA, 0.5 mM EGTA, 1% Triton X-100, 0.1% Sodium deoxycholate, 0.1% SDS) supplemented with 5 mM Na_3_VO_4_, 10 mM NaPP_i_, 100 mM NaF, 500 μM PMSF, and 5 mg/ml eComplete Mini protease inhibitor tablet (Roche) [33]. Western analyses was also used to examinee lysates from SAOS-2 or UMR-106 cells collected after the indicated treatments. Cells were lysed using RIPA buffer on ice. Equal concentrations of the clarified protein from bone or cell lysate were resolved on a 10% SDS-PAGE gel and transferred to a nitrocellulose membrane. Subsequently, the membrane was blocked with 5% milk and the proteins were detected using primary antibodies against pSmad3 (gift from Dr. E. Leof), Smad3 (abcam ab28379), Sclerostin (Santa Cruz, S-19) and β-actin (Abcam, ab8226) and secondary antibodies tagged with an infrared fluorophore. Blots were imaged using a Licor infrared imaging system and are representative of at least 3 experiments.

### Statistical Analyses

Statistics were performed using GraphPad Prism 5. T-tests were used to compare the differences between groups of normally distributed data. The Mann-Whitney nonparametric tests were used for non-normally distributed data. Significance of comparisons is defined by p-values equal to or less than 0.05.

## Results

### Load represses TGFβ signaling through Smad2/3

**Figure 1:**
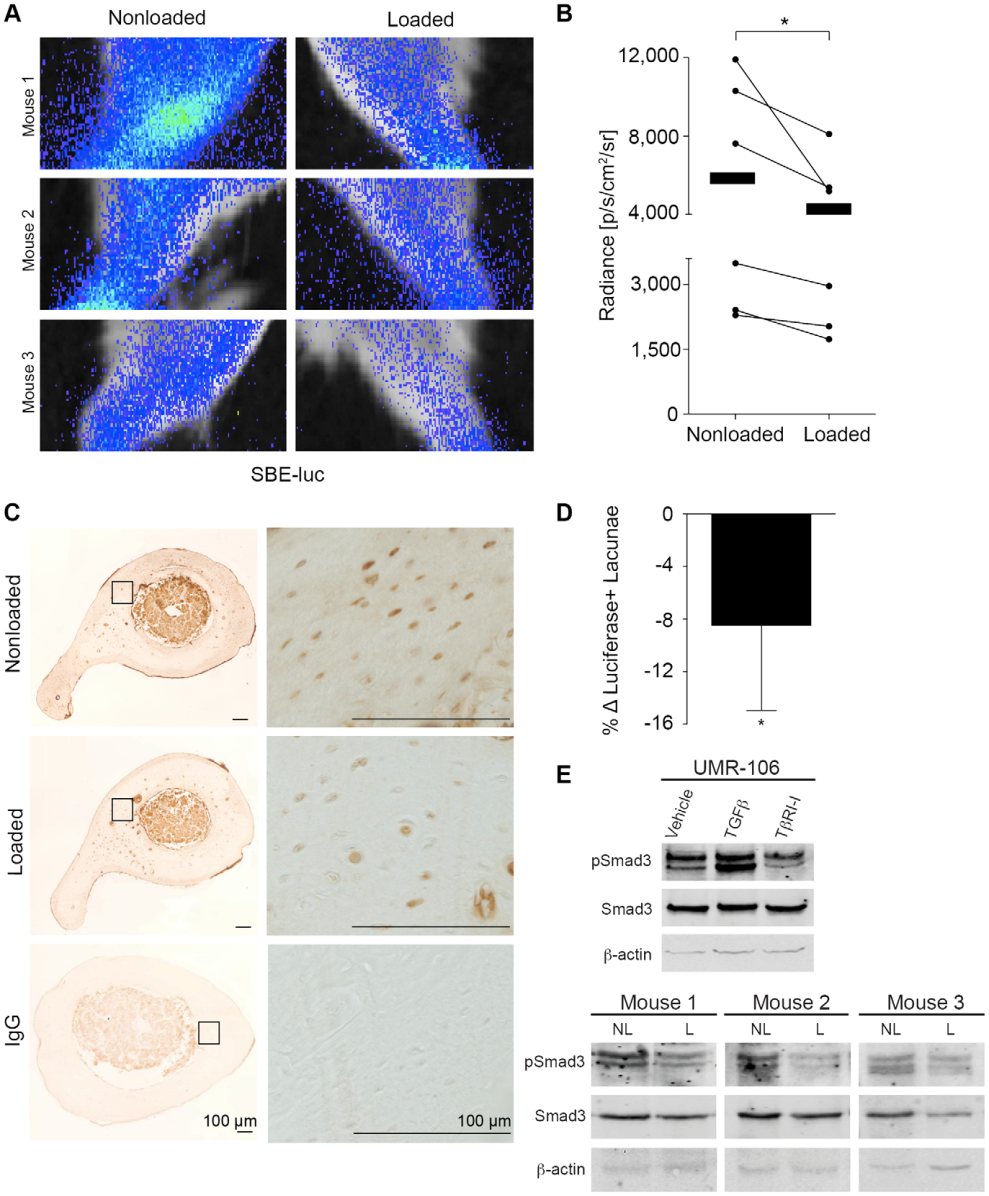
Mechanical load represses transactivation by Smad2/3 in tibial osteocytes. Images of luciferase activity in three individual SBE-luciferase mice 5h after *in vivo* loading reveals less Smad2/3-mediated transactivation in the loaded tibiae compared to nonloaded tibiae. Bioluminescence imaging and quantitation (A, B) show consistent reductions in luminescence in the loaded tibiae as opposed to the nonloaded tibiae. Radiance measurements from the nonloaded and loaded limb of the same animal are denoted by a line connecting the dots; black bars indicate the average radiance from nonloaded and loaded tibiae of 6 mice. Immunostaining for luciferase (C) in sections of tibial cortical bone and quantitation of luciferase-expressing osteocytes (D) reveal a decrease in luciferase-positive osteocytes in the loaded tibiae compared to the nonloaded control. UMR-106 whole cell lysates (E, upper panel) and tibial cortical bone lysates (E, lower panel) were evaluated for the level of phosphorylated Smad3, total Smad3, and β-actin by Western analysis. UMR-106 cells were harvested 2 h after treatment with vehicle (DMSO), TGFβ1 (5 ng/ml), or an inhibitor of the TGFβ type 1 receptor, Alk5 (TβRI-I, SB431542). Tibiae were harvested 3h after loading (n = 4 mice). Loaded tibiae (L) were compared to the nonloaded tibiae (NL) from the same mouse. (* p<0.05).

We used the murine one-limb loading model [29] to evaluate the role of TGFβ in load-induced bone formation. Mechanical stimulation was applied to one tibia of anesthetized SBE-Luc transgenic mice, which express luciferase under control of a Smad2/3-responsive synthetic promoter [30] [34]. By assessing the functional outcome of Smad2/3 activity, this approach evaluates the net effect of mechanical load on canonical TGFβ signaling – whether those effects occur at the ligand, receptor, or effector levels. Five hours after loading, *in vivo* bioluminescent imaging revealed that Smad2/3 reporter activity was consistently reduced in the loaded limb relative to the nonloaded limb of the same mouse (Figure 1A). Loading significantly repressed TGFβ-mediated Smad2/3 reporter activity by an average of 40% (Figure 1B). Immunolocalization of luciferase expression confirms that the load-mediated repression of Smad2/3 activity occurs in osteocytes within the cortical bone. In addition to generalized reduction in luciferase staining of cortical bone (Figure 1C), the number of luciferase positive osteocyte lacunae was significantly reduced in response to mechanical loading (Figure 1D). To further examine the mechanism by which load represses Smad2/3-mediated transactivation, we compared the effect of *in vivo* mechanical loading to the effect of *in vitro* TGFβ stimulation or inhibition on Smad3 phosphorylation. The level of phosphorylated Smad3 in the loaded cortical bone was consistently reduced relative to that in the nonloaded bone of the same animal (Figure 1E, lower panel). The magnitude of this load-dependent effect is comparable to that achieved in UMR-106 cells treated with an inhibitor of the TGFβ type I receptor (Figure 1E, upper panel). Therefore, the phosphorylation and activity of the key TGFβ effector, Smad3, is rapidly and significantly reduced in osteocytes following mechanical loading of the limb.

### The anabolic response of bone to mechanical load requires TGFβ signaling

**Figure 2:**
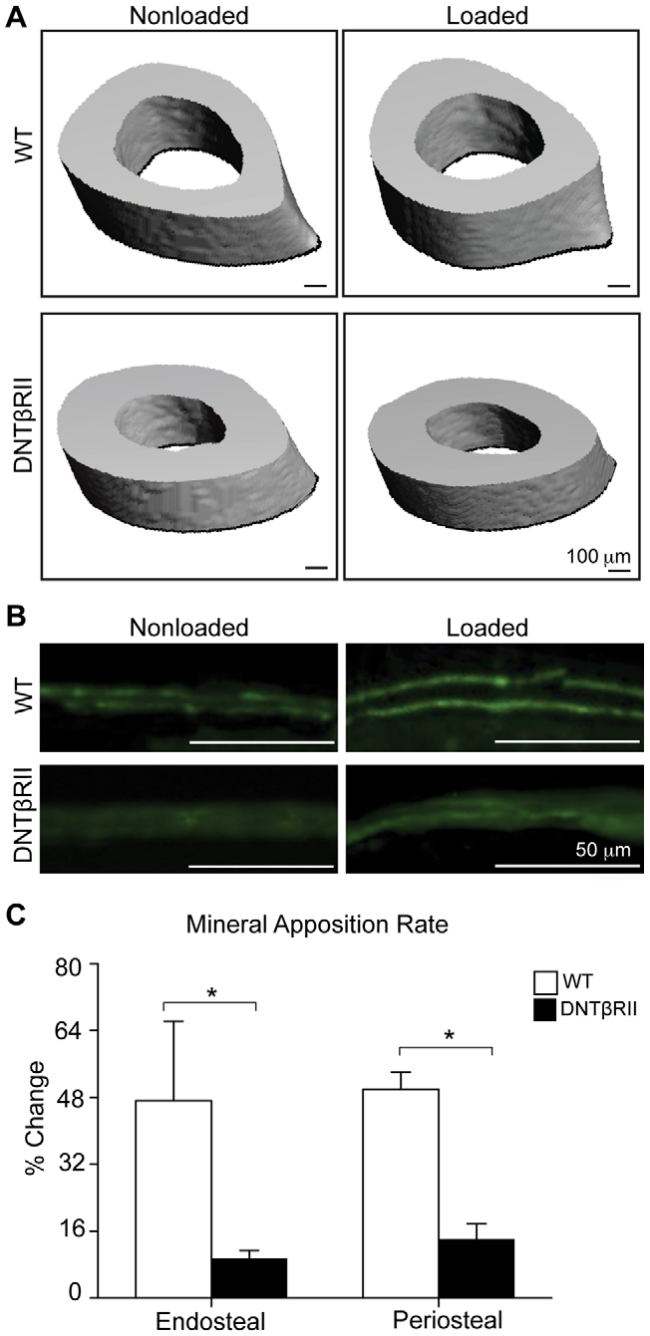
TGFβ signaling is required for load-induced bone formation. Micro-computed tomography images of loaded and nonloaded WT and DNTβRII tibiae (A) show that loading increases cortical bone thickness of WT bone more than DNTβRII bone. The overall fluorochrome intake was reduced in the DNTβRII mice despite a slightly increased basal mineral apposition rate in DNTβRII mice relative to WT (B). Results of dynamic bone histomorphometry are consistent with micro-CT, showing that the relative load-mediated increase in bone mineral apposition rate is significantly lower in DNTβRII tibiae than in WT (C) (* p<0.05).

We next examined whether the mechanosensitive regulation of TGFβ is necessary for load-induced bone formation. Mechanical load was applied to one limb of DNTβRII mice that have impaired TGFβ signaling due to expression of a dominant negative TGFβ type II receptor under control of the osteocalcin promoter [28]. In wild-type littermates, 5 days of mechanical loading stimulates a 19% increase in trabecular bone volume fraction (BV/TV) relative to the nonloaded limb (Table 1), similar to other studies [35], [36]. However, mechanical stimulation in DNTβRII mice only increases bone formation by 9%. Mechanical load also failed to stimulate the same magnitude of increase in DNTβRII trabecular connectivity (Tb. Conn), cortical thickness (Cort. Th.), and moment of inertia (MOI) compared to wild-type animals (Figure 2A, Table 1). Impaired TGFβ signaling in DNTβRII mice compromises load-induced bone formation at both the periosteal and endosteal surfaces, as determined by the reduced fluorochrome intensity in the loaded DNTβRII bone compared to the loaded WT bone (Figure 2B). Despite a slightly increased basal mineral apposition rate (MAR) in DNTβRII mice, relative to WT, mechanical load was unable to further stimulate a significant increase in the DNTβRII MAR. In contrast, mechanical load stimulated large (>40%) and significant increases in WT MAR relative to the nonloaded WT limb (Figure 2C). These results demonstrate that the anabolic effect of mechanical load on bone formation requires an active TβRII-dependent signaling pathway.

**Table 1:** Quantitative measures of micro-computed tomography analyses.

Parameters	WT			DNTβRII		
	*Nonloaded*	*Loaded*	*% Change*	*Nonloaded*	*Loaded*	*% Change*
*BV/TV*	0.180+/−0.082	0.215+/−0.13*	19.4+/−5.5%	0.280+/−0.062	0.303+/−0.091*	**8.92+/−6.6%**
*Tb. Conn. [1/mm^3^]*	30.5+/−5.5	33.3+/−6.7*	9.20+/−7.3%	36.8+/−6.5	37.8+/−8.7	**2.90+/−3.1%**
*Cort. Th. [mm]*	0.218+/−0.11	0.249+/−0.096*	14.0+/−4.6%	0.265+/−0.13	0.287+/−0.15*	**8.30+/−3.4%**
*MOI [1/mm^4^]*	0.0530+/−0.022	0.0640+/−0.024*	12.0+/−4.3%	0.0665+/−0.014	0.0731+/−0.019*	**9.00+/−3.2%**

Values represent means and standard deviations for n = 5 mice. An asterisk (*) represents significant differences between loaded and nonloaded bones (p<0.05). Bolded values indicate parameters that differ significantly between WT and DNTβRII mice (p<0.05).

### TGFβ sensitivity is required for regulation of Sclerostin by mechanical load

**Figure 3:**
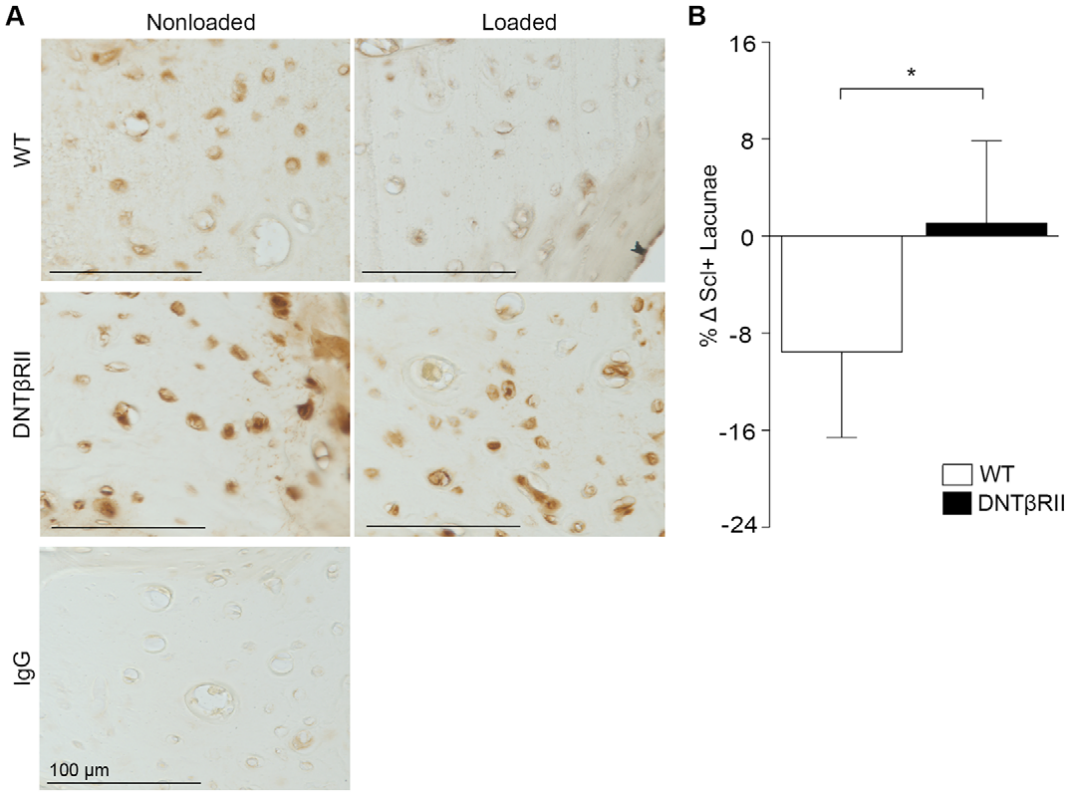
Load-sensitive Sclerostin regulation requires TGFβ signaling. Immunohistochemistry shows reduced Sclerostin expression between the loaded and nonloaded tibiae of WT mice, but no difference in Sclerostin expression in tibiae of DNTβRII mice (A). Control IgG used in the primary step of immunohistochemistry verifies the specificity of the SOST staining. Quantitation of Sclerostin-positive osteocytes in the tibiae confirm an 8% reduction in Sclerostin expression in loaded WT, but not in DNTβRII tibiae (B). (* p<0.05).

Since the repression of *SOST*/Sclerostin by mechanical load is a key event in bone anabolism [6], [37], we sought to determine if *SOST* regulation requires an intact TGFβ signaling pathway. We evaluated the effect of mechanical load on Sclerostin expression in DNTβRII mice. Following 5 days of loading, Sclerostin expression in wild-type cortical bone is reduced (Figure 3A). Specifically, the percentage of Sclerostin positive lacunae decreases by 9% following mechanical loading of the tibia (Figure 3B). However, in DNTβRII bone, the application of mechanical load produced no distinguishable difference in Sclerostin expression between the loaded and control limbs (Figures 3A, 3B), suggesting that the load-mediated regulation of Sclerostin requires sensitivity to TGFβ.

### TGFβ signaling through Smad3 induces SOST expression

**Figure 4:**
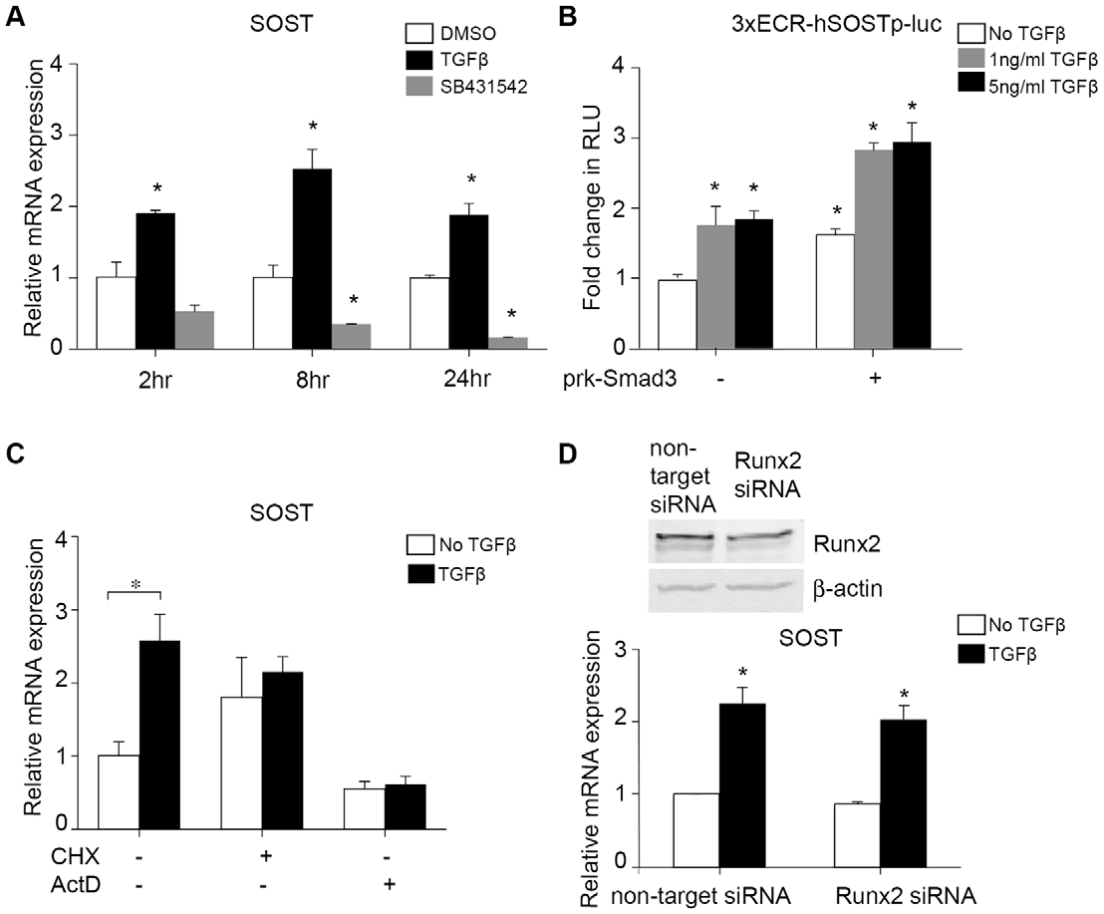
TGFβ induces *SOST* expression through Smad3. Treating UMR-106 cells with TGFβ1 (5 ng/ml) for 2, 8, or 24 h results in an increase in *SOST* mRNA, while inhibiting TGFβ signaling with SB431542 results in a decrease in *SOST* expression (A). Smad3 overexpression with pRK5-Smad3 induces the *SOST* promoter-reporter construct, 3XECR-hSOSTpLuc, in a TGFβ dose-dependent manner within 24 h of TGFβ treatment (B). Blocking translation with cycloheximide (CHX) or transcription with actinomycin-D (ActD) for 2 h prevents TGFβ (5 ng/ml) induction of *SOST* expression (C). siRNA mediated knockdown of *Runx2* did not prevent TGFβ (5 ng/ml) induction of *SOST* expression (D). (For panel C, * represents p<0.05 computed by comparing samples with added TGFβ to samples without added TGFβ in each treatment group; for all other panels, * p<0.05 computed by comparing samples to untreated cells).

To examine mechanisms by which TGFβ sensitivity is required to regulate *SOST*, we first evaluated the effect of TGFβ on *SOST* mRNA levels in UMR-106 osteosarcoma cells. TGFβ rapidly induces *SOST* mRNA expression, with maximal induction following 8 h of treatment (Figure 4A). Conversely, *SOST* mRNA expression is repressed by a specific inhibitor of the TGFβ type I receptor (TβRI, Alk5) inhibitor SB431542 (Figures 4A). Consequently, TGFβ rapidly induces *SOST* expression *in vitro*.

Since mechanical load represses TGFβ signaling through Smad2/3 in osteocytes (Figure 1), as well as *SOST* expression [6], we hypothesized that TGFβ induction of *SOST* is Smad2/3-dependent. Even without exogenous TGFβ, cotransfected Smad3 was sufficient to activate a promoter-reporter construct that expresses luciferase under control of the human *SOST* promoter and 3 copies of a previously identified *SOST*-regulatory enhancer sequence (ECR5) (Figure 4B). Cotransfected Smad3 further enhanced the TGFβ-inducibility of this construct.

### TGFβ/Smad3 induction of SOST is indirect and insensitive to Runx2

Though Smad3 increases *SOST*-reporter activity, these effects are indirect. As expected, incubation of UMR-106 cells with actinomycin D, an inhibitor of transcription, blocks TGFβ-inducible *SOST* mRNA expression (Figure 4C). However, TGFβ-inducible *SOST* mRNA expression is also completely abrogated in the presence of cycloheximide, an inhibitor of translation, even at the earliest 2 h time point (Figure 4C). These data suggest that TGFβ-inducible *SOST* expression occurs through an indirect Smad3-mediated pathway.

TGFβ and Smad3 regulate the expression and activity of the osteoblast transcription factor Runx2 [20]. Since *SOST* is a Runx2-target gene [14], we sought to determine if Runx2 was required for the indirect TGFβ/Smad3-dependent regulation of *SOST* expression. *Runx2*-targetting siRNA yielded a 35–80% decrease in *Runx2* mRNA levels in UMR-106 and SAOS cells (not shown), with a corresponding reduction in Runx2 protein expression (Figure 4D). This treatment was sufficient to reduce the expression of Runx2-inducible RANKL by 35% (not shown). However, even with reduced levels of *Runx2*, the TGFβ-mediated induction of *SOST* mRNA was intact (Figure 4D), demonstrating that TGFβ-mediated induction of *SOST* is insensitive to the level of Runx2 activity. Together these findings show that TGFβ regulates SOST mRNA expression indirectly through a Smad3-dependent, Runx2-insensitive mechanism.

## Discussion

**Figure 5:**
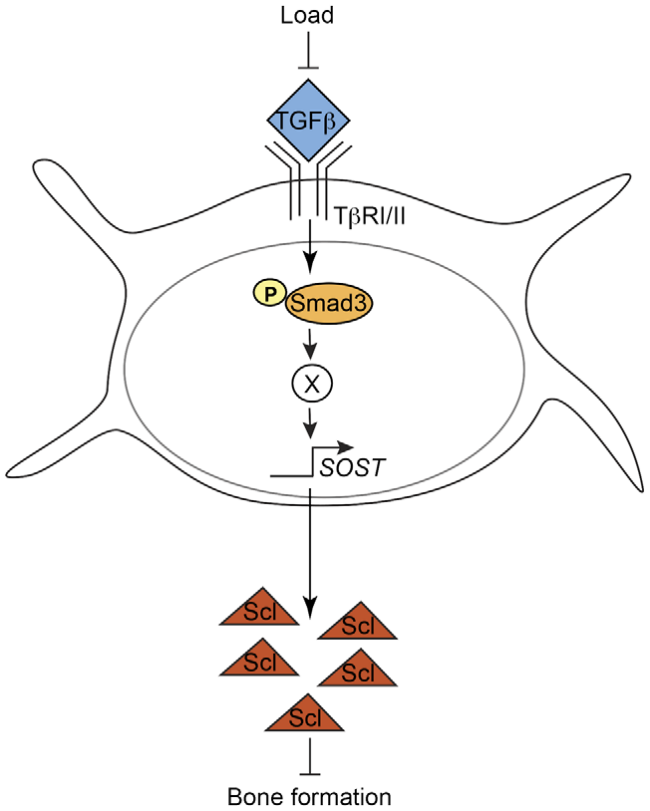
The role of TGFβ signaling in load-induced bone formation. Load represses TGFβ signaling through Smad2/3 in osteocytes, which is required for the activation of SOST expression through an indirect, Runx2-independent mechanism. Loss of function mutations in the TGFβ type II receptor impair load-mediated repression of SOST and new bone formation.

Here, we report that mechanical load represses TGFβ activity, which is required for load-induced bone formation and the regulation of Sclerostin, an inhibitor of bone anabolism (Figure 5). Loading of mice tibiae rapidly inhibits phosphorylation of Smad3, a TGFβ effector, and consequently represses Smad3 activity in osteocytes. TGFβ signals through Smad3 to indirectly stimulate *SOST* mRNA expression. Furthermore, intact TGFβ signaling is required for load to repress Sclerostin expression and induce bone formation. Taken together, our results demonstrate that TGFβ plays a critical role in the mechanosensitive regulation of Sclerostin and is required for the anabolic response of bone to mechanical load.

Several lines of evidence implicate TGFβ as a regulator of bone homeostasis. Many mouse models with mutations in components of the TGFβ pathway have altered bone mass and bone matrix material properties, resulting from disruption of the tightly controlled balance between osteoblast and osteoclast activity. For example, reducing TGFβ signaling in mice by expression of a dominant negative TβRII allele in the osteoblast lineage increased bone mass by indirect reduction in osteoclast activity [28]. In addition, mice deficient in Smad3 have lower bone mass but an increased bone matrix elastic modulus indicating the importance of TGFβ in the maintenance of bone mass and bone quality [38]. Furthermore, inhibiting TGFβ signaling with a chemical inhibitor of TβRI affects postnatal bone formation and bone quality [30]. Stimuli that shift bone metabolism, including mechanical load, exert their effects by acting on pathways that normally maintain homeostasis. The extent to which TGFβ mediates the anabolic effect of mechanical load on bone has not previously been shown. Here, we show that disrupting TGFβ sensitivity in osteoblasts prevents mechanical load from inducing bone formation. This suggests that mice with mutations in the TGFβ pathway uncouple the normal regulation of bone mass from stimuli such as mechanical load; likely contributing to the high or low bone mass phenotypes observed in a several mouse models and human diseases in which TGFβ signaling is deregulated.

Mechanical load regulates TGFβ signaling at multiple levels. Cell-generated tensile stress induces the activation of the latent TGFβ ligand by myocytes [22]. *In vitro* fluid flow stimulates the expression of TGFβ1 mRNA in osteoblast cells [21]. Also, loading of the rat ulna induces TGFβ1 mRNA expression in the periosteal bone within 4h [39]. Still unclear, however, is the net functional effect of these mechanosensitive changes at multiple hierarchical levels of the TGFβ pathway. Unexpectedly, our results show that mechanical load rapidly inhibits Smad3 phosphorylation and, thus, represses the activity of key TGFβ effectors, Smad2 and Smad3, in osteocytes. Therefore, mechanical stimulation regulates the TGFβ pathway at the transcriptional level, as described above, as well as through post-transcriptional regulation of Smad2/3 activity.

TGFβ can be added to the list of pathways found to be mechanosensitive in bone that also regulate Sclerostin. PGE_2_ regulates both osteoblast and osteoclast activities and is produced within 5 mins of mechanical stimulation [40]. Once mechanically stimulated, PGE_2_ signals through the EP4 receptor to repress *SOST* transcription in osteoblast cells [41]. Several lines of evidence implicate PTH in load regulation of SOST activity. First, PTH levels are elevated in serum during high impact exercises such as running [3]. Second, *SOST* overexpression desensitizes mice to PTH-induced bone formation, suggesting that *SOST* acts as a downstream target of PTH [42]–[43]. Third, PTH represses *SOST* expression by inhibiting MEF2 transcriptional activity at the *SOST* enhancer [12] [11]. Some gaps in the connection between load, PGE_2_ and PTH, and *SOST* remains to be filled.

Here we find that load fails to repress Sclerostin expression by osteocytes or new bone formation in the tibia of DNTβRII mice. Along with the mechanosensitive regulation of Smad2/3 function in osteocytes, these findings establish a clear link between mechanical load, TGFβ signaling, *SOST* levels, and new bone formation. It is important to note that modest bone formation was detected in the loaded tibia of DNTβRII mice (3–9%). Clearly, multiple other pathways in addition to TGFβ cooperate to regulate bone anabolism following mechanical load. Since the PTH and TGFβ pathways induce desensitization of one another via receptor internalization [23], some of the PTH-dependent effects may occur through TGFβ-dependent mechanisms. *SOST* may be a target of crosstalk between the PTH and TGFβ pathways. For example, the *SOST* regulatory transcription factor, MEF2, is repressed by PTH in osteoblasts [12] as well as by TGFβ/Smad3 in myocytes [44]. Furthermore, MEF2 is required for the activation of *SOST* transcription by TGFβ in osteogenic cells [45]. Thus, MEF2 may be a common target of both PTH and TGFβ and a point of convergence in their regulation of *SOST* and load-induced bone formation. Although mechanical load represses Smad2/3 activity in osteocytes, it remains possible that load regulation of *SOST* through TGFβ occurs indirectly through the effects of TGFβ on osteoblasts since DNTβRII is not expressed exclusively in osteocytes but also in osteoblasts. Further studies using osteocyte-specific mutations would clarify the precise role of TGFβ in each cell type in the response to load.

In conclusion, we find that load represses Smad3 activity in osteocytes and signals through the TGFβ pathway to maintain bone homeostasis. TGFβ indirectly induces *SOST* expression, which is mediated by Smad3. Although Runx2 was previously shown to regulate *SOST* expression [14], the TGFβ and Smad3-dependent induction of *SOST* is insensitive to the level of Runx2 activity. Finally, our data reveal a novel mechanism in load regulation of bone formation, which could provide insights for treating bone diseases such as osteoporosis.
